# Characteristics of progressive multifocal leukoencephalopathy in hematological malignancies, With reference to HIV-infected patients

**DOI:** 10.1007/s12185-026-04256-5

**Published:** 2026-07-24

**Authors:** Emi Yokoyama, Taisuke Harada, Tomoyuki Endo, Hideki Goto, Junichi Sugita, Masayuki Aiba, Mutsumi Takahata, Takuto Miyagishima, Hajime Senjo, Satoshi Iyama, Shuichiro Takahashi, Masayo Yamamoto, Motohiro Shindo, Kazuya Sato, Junichi Hashiguchi, Tatsuo Oyake, Takanori Teshima

**Affiliations:** 1 Blood Disorders Center, Aiiku Hospital, Sapporo, Japan; 2 Department of Diagnostic Imaging, Hokkaido University Hospital, Sapporo, Japan; 3 Department of Infection Control and Prevention, Hokkaido University Hospital, Sapporo, Japan; 4 Department of Hematology, Hokkaido University Hospital, Sapporo, Japan; 5 Department of Hematology, Sapporo Hokuyu Hospital, Sapporo, Japan; 6 Department of Hematology, Sapporo Kosei General Hospital, Sapporo, Japan; 7 Department of Hematology, Kushiro Rosai Hospital, Kushiro, Japan; 8 Department of Hematology, Sapporo Medical University Hospital, Sapporo, Japan; 9 Division of Hematology, Department of Internal Medicine, Asahikawa Medical University, Asahikawa, Japan; 10 Department of Hematology/Oncology, Asahikawa Kosei General Hospital, Asahikawa, Japan; 11 Department of Hematology, Kitami Red Cross Hospital, Kitami, Japan; 12 Division of Hematology and Oncology, Department of Internal Medicine, Iwate Medical University School of Medicine, Yahaba, Japan; 13 Department of Hematology, Faculty of Medicine, Hokkaido University, Sapporo, Japan

**Keywords:** Progressive multifocal leukoencephalopathy, Hematological malignancies, Human immunodeficiency virus infection

## Abstract

Progressive multifocal leukoencephalopathy (PML) is a rare opportunistic infection of the central nervous system (CNS) observed in immunocompromised individuals. We retrospectively evaluated the clinical courses of PML in patients with hematological malignancies at North Japan Hematology Study Group institutions, using patients with human immunodeficiency virus (HIV) infection as a reference. Eight PML patients had hematological malignancies and 9 had HIV infection. CD4 + T cell counts were markedly low in patients with hematological malignancies (median 168/μl, range 48.4–330/μl) and did not differ significantly from those in patients with HIV infection (median 28/μl, range 8–225.4/μl). IgG levels were significantly lower in patients with hematological malignancies (median 643.5 mg/dL, range 205–2478 mg/dL) than in patients with with HIV infection (median 1691.5 mg/dL, range 1304–3014 mg/dL) (*p* < 0.01). Almost all patients with hematological malignancies died after progression of PML (median time from onset to death, 136 days). In contrast, seven patients with HIV infection were alive at the end of follow-up. The incidence of PML in patients with hematological malignancies may increase with the introduction of new antibody drugs and cellular therapies in future. Considering the poor prognosis, greater caution is warranted regarding this serious complication.

## Introduction

Progressive multifocal leukoencephalopathy (PML) is an opportunistic infection of the central nervous system (CNS) caused by the John Cunningham polyomavirus (JCV). In patients with PML, JCV infects oligodendrocytes and induces multifocal demyelination in the cerebrum, cerebellum, and brain stem [1–3]. The symptoms of PML include variable neurological dysfunctions depending on the affected sites. Due to its rarity, the symptoms of PML are often misdiagnosed as cerebral infarction, infectious encephalitis, or CNS invasion of malignancies, leading to a delayed diagnosis.

PML was initially an extremely rare disease and was first described as a demyelinating disease in a patient with chronic lymphocytic leukemia and Hodgkin’s disease in 1958 [4]. After the global human immunodeficiency virus (HIV) pandemic began in 1981, PML was occasionally observed among patients with acquired immunodeficiency syndrome (AIDS) [5]. The incidence of PML in patients with HIV infection significantly decreased after the introduction of antiretroviral therapy (ART). On the other hand, iatrogenic immune deficiency has become the leading cause of PML with the development of various immunosuppressive and immunomodulatory drugs mainly used for autoimmune diseases and hematological disorders [6–8].

The characteristics of PML in patients with hematological malignancies are not fully elucidated, and their differences from PML in patients with HIV infection have not been assessed. To address this issue, we retrospectively investigated the characteristics and clinical courses of PML in patients with hematological malignancies, using those in patients with HIV infection as a reference, among institutions of the North Japan Hematology Study Group (NJHSG).

## Methods

### Study design and data source

This is a retrospective, multi-institutional study using data retrieved from the clinical records of patients treated in the institutions of the NJHSG. This study was approved by the Institutional Review Board of Aiiku Hospital and the other participating institutions. Because this is a non-interventional, retrospective study using anonymized clinical data, the requirement for informed consent from each patient was waived and opt-out information was provided on the websites of the participating institutions.

### Study subjects

Patients with hematological malignancies or HIV infection who were diagnosed with PML from January 1, 2010, to August 31, 2025, at the participating institutions were included.

### Diagnosis of PML

**Fig. 1:**
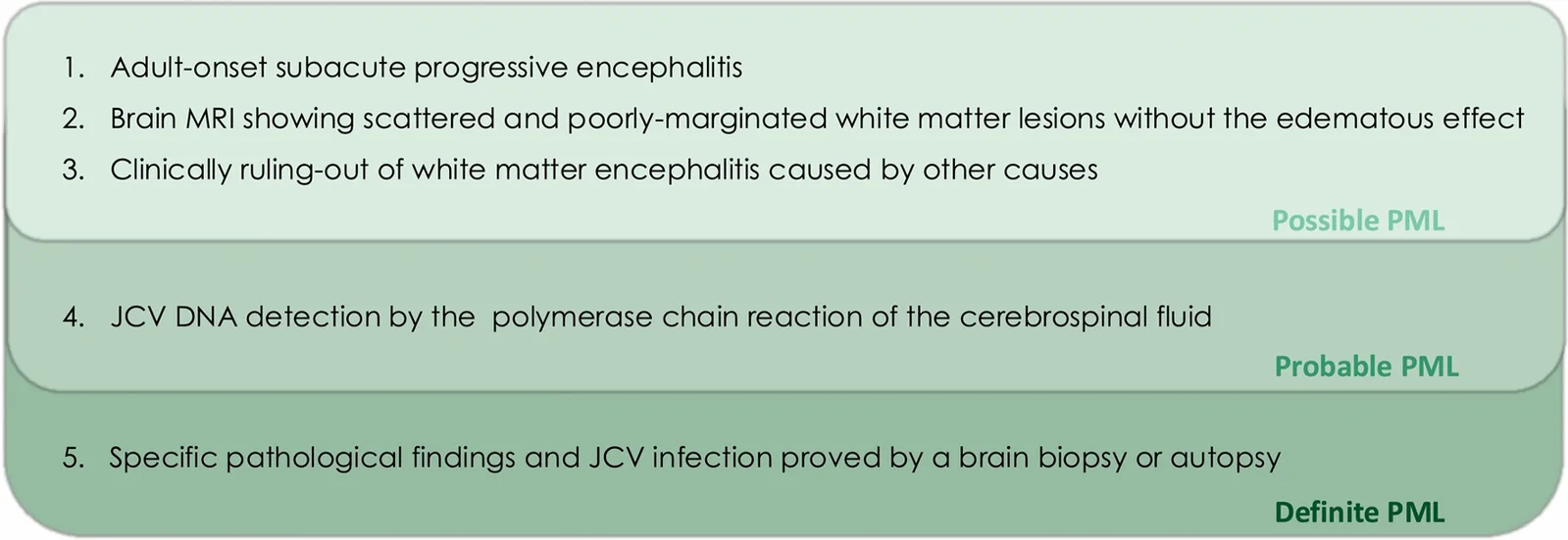
Categories of PML diagnosis (the Japanese Ministry of Health, Labour and Welfare)

The diagnosis of PML was categorized into “definite,” “probable,” and “possible” PML according to the classification of the Japanese Ministry of Health, Labour and Welfare (Fig. 1). For the diagnosis of “definite PML,” specific pathological findings and JCV infection proven by a brain biopsy or autopsy were required. For “probable PML,” the detection of JCV DNA by polymerase chain reaction (PCR) in the cerebrospinal fluid (CSF) was needed. Even if JCV DNA was not tested or detected in the CSF, adult-onset subacute encephalitis with scattered white matter irregular lesions without edematous effect can be diagnosed as “possible PML” by ruling out leukoencephalopathy due to other causes.

### Assessment of immunological state and statistics

CD4 + T cell counts and immunoglobulin G (IgG) levels in the peripheral blood at the diagnosis of PML (within 4 weeks before or after diagnosis) were assessed. The Mann–Whitney U test was used to compare these variables between patients with hematological malignancies and those with HIV infection. The statistical analysis was performed using EZR software [9]. Statistical significance was defined as a *p*-value of less than 0.01. In addition, for patients with HIV infection treated with ART, CD4 + T cell counts at 3, 6, 9, and 12 months after the PML diagnosis were assessed, where available.

## Results

### Patient characteristics

#### PML in patients with hematological malignancies

We confirmed eight cases (male, n = 3: female, n = 5: median age at diagnosis of PML: 72 [range 58–95] years) of PML in patients with hematological malignancies (Cases M1-M8, Table 1). Hematological malignancies included diffuse large B cell lymphoma (DLBCL: n = 3), follicular lymphoma (FL: n = 3), angioimmunoblastic T cell lymphoma (AITL: n = 1), and multiple myeloma (MM: n = 1). All patients with DLBCL had received rituximab plus cyclophosphamide, doxorubicin, vincristine, and prednisolone (R-CHOP) therapy for the first-line treatment (Cases M1-M3). Case M1 was primary refractory and proceeded to chimeric antigen receptor T (CAR-T) cell therapy after three lines of chemotherapy. The patient developed PML 227 days after the initiation of CAR-T cell therapy. Case M2 (DLBCL) relapsed after six courses of R-CHOP therapy. The patient developed neurocognitive dysfunction with a white matter lesion on brain magnetic resonance imaging (MRI).

**Table 1:** Patient characteristics and the diagnosis of PML in patients with hematological malignancies. DLBCL, diffuse large B cell lymphoma; FL, follicular lymphoma; AITL, angioimmunoblastic lymphoma; MM, multiple myeloma; R-CHOP, rituximab + cyclophosphamide + doxorubicin + vincristine + prednisolone; R-DA-EPOCH; rituximab + dose-adjusted etoposide + prednisolone + vincristine + cyclophosphamide + doxorubicin; DeVIC, dexamethasone + etoposide + ifosfamide + carboplatin; CAR-T, chimeric antigen receptor T cell; IFRT, involved field radiation therapy; Hd-MTX, high-dose methotrexate; GB, Obinutuzumab + bendamustine; THP-COP, pirarubicin + cyclophosphamide + vincristine + prednisolone; BV, brentuximab vedotin; CHASE, cyclophosphamide + cytosine arabinoside + etoposide + dexamethasone; CBT, cord blood transplantation; MP, melphalan + prednisolone; RT, radiation therapy; BD, bortezomib + dexamethasone; LD, lenalidomide + dexamethasone; EPd, elotuzumab + pomalidomide + dexamethasone

No	Age	Sex	Disease	Treatment history of the disease	PML onset from the last treatment (days)	PCR analysis (CSF^†^)	The viral load of the CSF (copy/ml)	Brain biopsy or autopsy	The category of diagnosis	From onset to the diagnosis (days)
M1	58	M	DLBCL	R-CHOP, R-DA-EPOCH, DeVIC, CAR-T	227	o	156,000	−	Probable	2
M2	80	F	DLBCL	R-CHOP, IFRT, R-EPOCH, Hd-MTX	During treatment	−	−	−	Possible	33
M3	74	M	DLBCL	R−CHOP	During treatment	−	−	−	Possible	−
M4	70	F	FL	GB	487	o	90,000	−	Probable	86
M5	63	F	FL	R-CHOP	5534	o	54,930	o	Definite	91
M6	95	M	FL	GB	33	o	200,000	−	Probable	3
M7	59	F	AITL	THP-COP, BV, CHASE, romidepsin, DA-EPOCH, CBT (FM140 + TBI4Gy)	588	o	300,000	−	Probable	49
M8	74	F	MM	MP, RT, BD, LD, EPd	During treatment	o	500,000	−	Probable	18

^†^CSF, cerebrospinal fluid

^‡^ART, antiretroviral therapy

^§^PCR, polymerase chain reaction

^¶^HIV, human immunodeficiency virus

after three courses of salvage treatment with rituximab plus etoposide, doxorubicin, vincristine, cyclophosphamide, and prednisolone (R-EPOCH). CNS invasion of malignant lymphoma was initially suspected, and high-dose methotrexate therapy was initiated. However, the white matter lesion deteriorated, leading to the suspicion of PML. Case M3 developed symptoms of PML after four courses of R-CHOP. A patient with FL (Case M4) had received six courses of obinutuzumab plus bendamustine (GB) treatment and developed PML 487 days after the last treatment. Case M5 had relapsed FL 15 years after the last treatment with six courses of R-CHOP. The brain lesions appeared at the same time as the lymphoma relapse. The lesions were initially considered to be old infarctions or lymphoma invasion of the CNS. After two courses of the salvage treatment with rituximab plus bendamustine, the brain lesion deteriorated, leading to the diagnosis of PML. A 95-year-old patient with FL (Case M6) had received two courses of GB treatment at the onset of PML after watchful waiting for 17 months from the diagnosis of FL. The patient with AITL (Case M7) had received cord blood transplantation (CBT) after five lines of chemotherapy. She developed acute graft-versus-host disease (GVHD) (grade II, gut stage 1) and immunosuppressive therapy with prednisolone and anti-thymocyte globulin was initiated. Acute GVHD resolved with these treatments. However, systemic steroid administration was restarted due to chronic GVHD of the gut, skin and oral cavity on day + 228. She developed PML on day + 558 after CBT. Case M8 was a patient with heavily pre-treated MM. When the patient developed PML, a combination therapy with elotuzumab, pomalidomide and dexamethasone had been repeated for 28 cycles after three lines of previous chemotherapy.

#### PML in patients with HIV infection

We confirmed nine cases of PML in patients with HIV infection (Cases H1–H9, Table 2). All were male, and the median age was 43 (range 35–68) years. Almost all patients developed PML before (or at the same time as) the diagnosis of HIV infection, with PML serving as the AIDS-defining illness. Only Case H3 was diagnosed with PML during a workup after the diagnosis of HIV infection, in the absence of any neurological symptoms.

**Table 2:** Patient characteristics and the diagnosis of PML in patients with HIV infection

No	Age	Sex	The onset of PML	From the onset of PML to the diagnosis of HIV (days)	ART^‡^ for HIV	PCR^§^ analysis (CSF)	The viral load of the CSF (copy/ml)	Brain biopsy or autopsy	The category of diagnosis	From onset to the diagnosis (days)
H1	62	M	Before the diagnosis of HIV^¶^	88	o	o	321,000	o	Definite	95
H2	44	M	Before the diagnosis of HIV	192	o	o	Negative	−	Possible	199
H3	41	M	At the same time of the diagnosis of HIV	−	o	o	11,000	o	Definite	20
H4	68	M	Before the diagnosis of HIV	133	o	o	524,500	−	Probable	136
H5	38	M	At the same time of the diagnosis of HIV	−	o	o	7588	−	Probable	19
H6	43	M	Before the diagnosis of HIV	52	o	o	2593	−	Probable	58
H7	48	M	Before the diagnosis of HIV	365	o	o	1096	−	Probable	365
H8	39	M	At the same time of the diagnosis of HIV	−	o	o	2,377,600	−	Probable	38
H9	35	M	At the same time of the diagnosis of HIV	−	o	o	Negative	−	Possible	11

^†^CSF, cerebrospinal fluid

^‡^ART, antiretroviral therapy

^§^PCR, polymerase chain reaction

^¶^HIV, human immunodeficiency virus

### Diagnosis

#### PML in patients with hematological malignancies

One patient with FL (Case M5) was diagnosed with “definite PML” based on a brain biopsy (Table 1). Five cases (M1, M4, M6, M7, and M8) were diagnosed with “probable PML.” The viral load of JCV DNA in the CSF for each patient is shown in Table 1. Two patients with DLBCL (Cases M2 and M3) did not undergo CSF testing but were diagnosed with “possible PML” based on clinical symptoms and MRI findings. Case M3 was retrospectively diagnosed with “possible PML” after death. The median time from onset to the diagnosis of PML was 33 (range 2–91) days. In most cases, other CNS conditions (e.g., involvement of hematological malignancies, cerebral infarction, or other types of encephalitis) were suspected at the onset of neurological symptoms, and the diagnosis of PML was delayed.

#### PML in patients with HIV infection

Two cases (H1 and H3) were diagnosed with “definite PML” (Table 2). Five cases (H4-H8) were diagnosed with “probable PML.” In the remaining two cases (H2 and H9), CSF tests were negative for JCV, as well as for other bacterial, viral and fungal pathogens tested. These cases showed typical brain MRI findings of PML with white matter lesions. After discussion with neurologists and radiologists, they were clinically diagnosed with “possible PML.” The median time from onset to the diagnosis of PML was 76.5 (range 11–365) days. In some patients, the diagnosis of PML was significantly delayed due to misdiagnosis as other neurological diseases.

### MRI findings and clinical manifestations

**Fig. 2:**
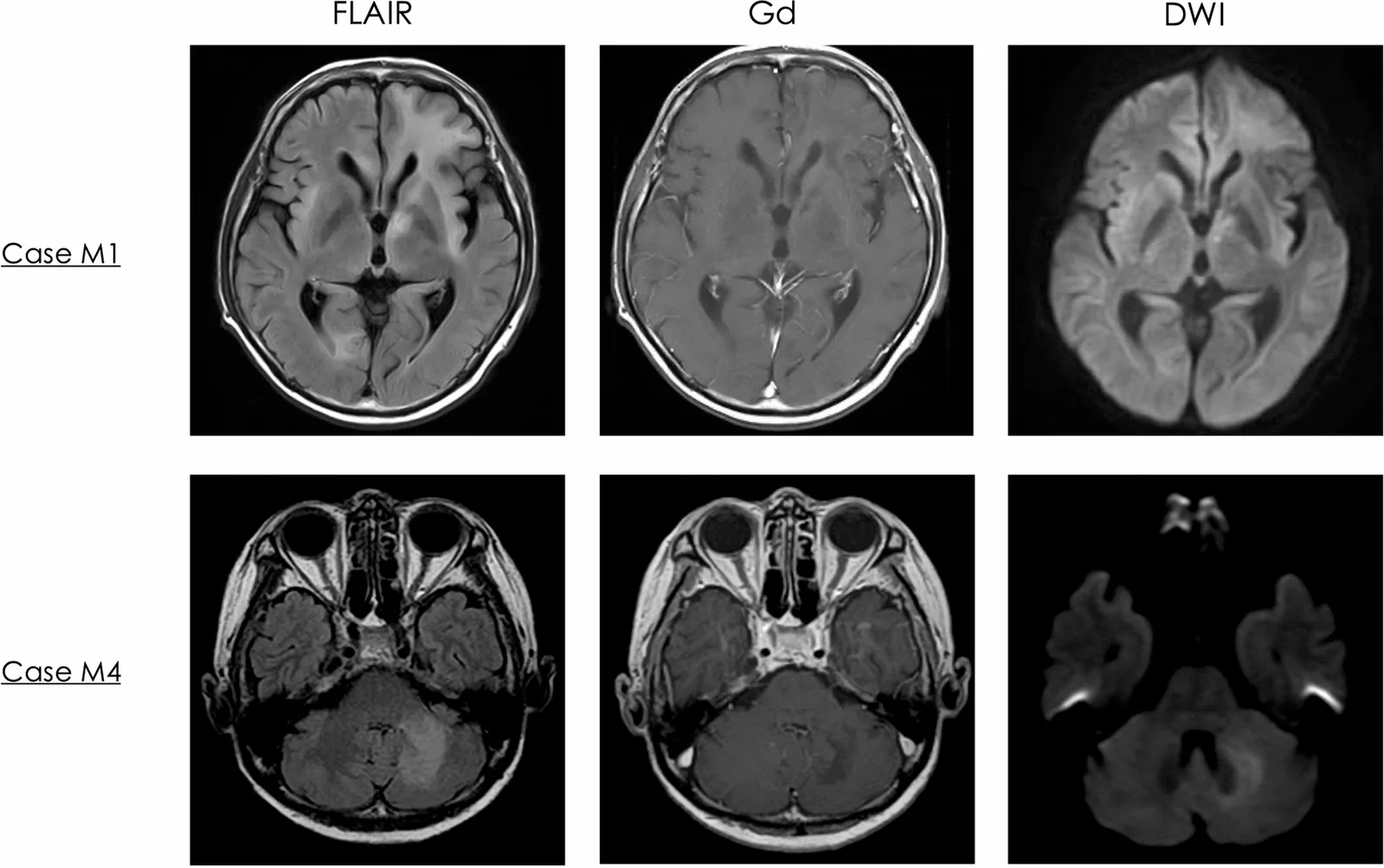
Representative brain MRI findings of PML in patients with hematological malignancies. Case M1 with DLBCL presented with PML lesions in the cerebrum, basal ganglia, thalamus, and brain stem. Case M4 with FL had a PML lesion in the cerebellum. Both cases showed poorly marginated white matter lesions without mass effect. These lesions exhibited high signal intensity on fluid-attenuated inversion recovery (FLAIR) images without gadolinium enhancement, and focal hyperintensity on diffusion-weighted images. FLAIR, fluid-attenuated inversion recovery; Gd, gadolinium; DWI, diffusion-weighted imaging

The specific characteristics of PML are the subacute progression of clinical symptoms and MRI findings of a poorly marginated, high-intensity lesion in the white matter on T2-weighted and fluid-attenuated inversion recovery imaging, without mass effect. Unlike malignant tumors, PML lesions are generally not enhanced by gadolinium. Representative MR images in this study are shown in Fig. 2. The affected sites at the diagnosis of each case are shown in Table 3 and B. Neurological manifestations included disturbance of consciousness, cognitive impairment, aphasia, gait disturbance, paresthesia, vertigo, and coordination impairment. Symptoms differed depending on the affected sites (Table 3 and 4) and changed over time with the progression of PML.

**Table 3:** Distribution and symptoms of PML and outcomes of PML in patients with hematological malignancies

No	Age	Sex	Disease	Sites of brain lesion	Symptoms	Treatment	Response to the treatment	outcome	Cause of death	From onset to death (days)	From the diagnosis to death (days)
M1	58	M	DLBCL	Cerebrum Basal ganglia Thalamus Brain stem	Disorientation Disturbance of consciousness	−	−	Dead	PML	113	111
M2	80	F	DLBCL	Cerebrum	Aphasia Semi-paralysis Gait disturbance	−	−	Dead	PML	154	121
M3	74	M	DLBCL	Cerebrum Basal ganglia Thalamus Corpus callosum	Cognitive dysfunction Gait disturbance Incontinence	−	−	Dead	PML	76	−
M4	70	F	FL	Cerebellum	Vertigo Coordination impairment	Mirtazapine	None	Dead	PML	178	92
M5	63	F	FL	Cerebrum	Immobility	−	−	Dead	PML	179	88
M6	95	M	FL	Cerebrum	Dysarthria Facial paralysis	−	−	Dead	PML	25	22
M7	59	F	AITL	Cerebrum Corpus callosum	Cognitive dysfunction Aphasia	−	−	Dead	PML	118	69
M8	74	F	MM	Cerebrum	Disorientation	Mefloquine Mirtazapine	None	Dead	Unknown	736	718

^†^IRIS, immune reconstitution inflammatory syndrome

^‡^CMV, cytomegalovirus

**Table 4:** Distribution and symptoms of PML and outcomes of PML in patients with HIV infection

No	Age	Sex	Sites of brain lesion	Symptoms	Treatment	IRIS^†^	Response to the treatment	Outcome	Cause of death	From onset to the end of follow-up (days)	From the diagnosis to the end of follow-up (days)
H1	62	M	Cerebrum Cerebellum	Aphasia Dysphasia Coordination impairment	Mefloquine ART	−	−	Dead	CML^‡^ colitis Sepsis DIC^§^	108	13
H2	44	M	Cerebrum Cerebellum Internal capsule Midbrain	Aphasia Coordination impairment	ART	−	o	Alive	−	564	365
H3	41	M	Cerebrum Cerebellum External capsule Midbrain Pons	Gait disturbance Dysarthria Coordination impairment	ART	−	o	Alive	−	3043	3023
H4	68	M	Cerebrum Cerebellum Midbrain Pons Internal capsule	Semi-paralysis Dysesthesia Hearing impairment Coordination impairment	ART	o	×	Dead	PML	208	72
H5	38	M	Cerebrum Cerebellum Pons	Semi-paralysis Dysarthria	ART	o	−	Alive	−	75	56
H6	43	M	Cerebrum Cerebellum Internal capsule	Gait disturbance Dysarthria	ART	−	o	Alive	−	229	171
H7	48	M	Cerebrum Cerebellum	Gait disturbance Dysarthria	ART	o	×	Alive	−	1261	896
H8	39	M	Cerebellum Brain stem	Dysesthesia Vertigo Nausea Diplopia	ART	−	×	Alive	−	476	438
H9	35	M	Cerebrum	Semi-paralysis	ART	−	o	Alive	−	844	833

^†^IRIS, immune reconstitution inflammatory syndrome

^‡^CMV, cytomegalovirus

^§^DIC, disseminated intravascular coagulation

### Immunological assessment

**Fig. 3:**
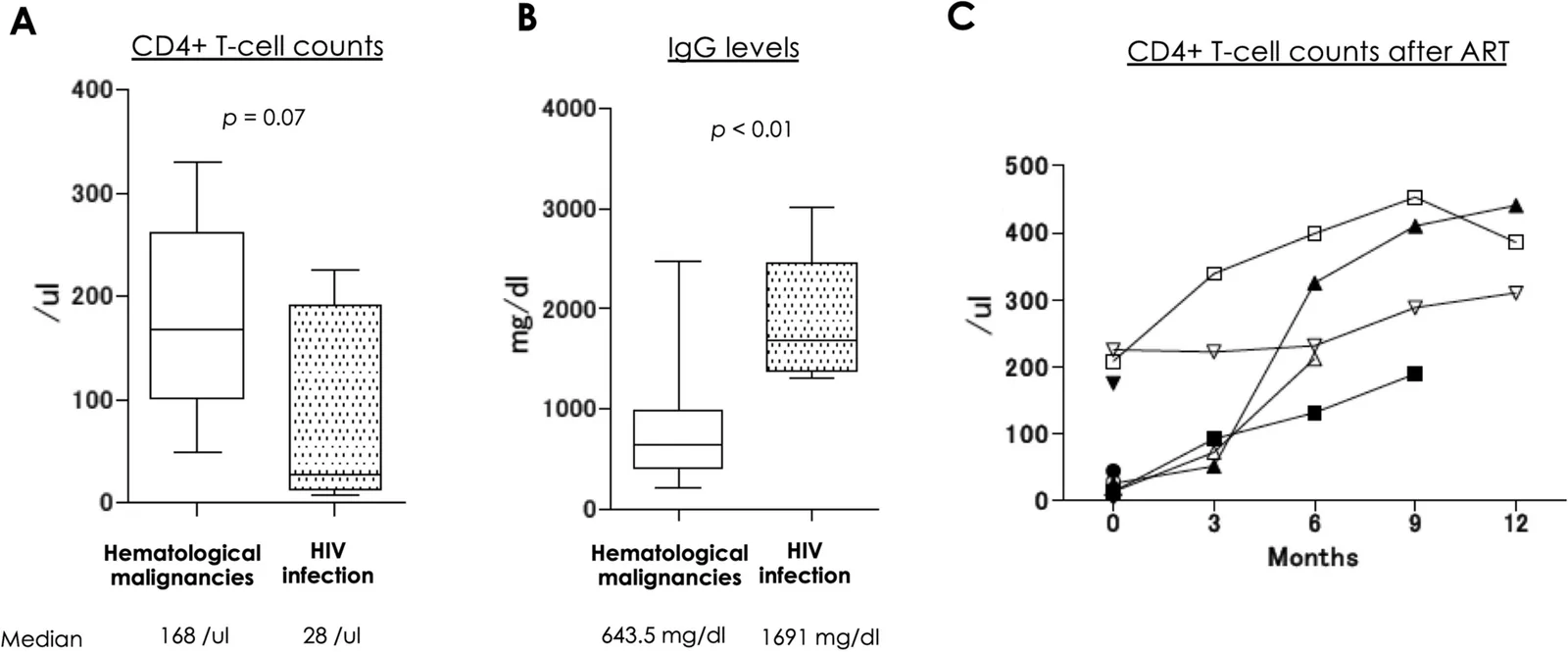
Immunological assessment at the diagnosis of PML. (**a**) CD4 + T cell counts were lower in patients with HIV infection than in those with hematological malignancies (median 28 vs. 168 /μl). (**b**) CD4 + T cell counts gradually increased after the initiation of ART in patients with HIV infection. (**c**) IgG levels in patients with hematological malignancies were significantly lower than those in patients with HIV infection (median, 643.5 vs. 1,691 mg/dl, *p* < 0.01)

CD4 + T cell counts were assessed in six of eight patients with hematological malignancies and all nine patients with HIV infection. CD4 + T cell counts in HIV patients were very low, as expected (median 28 /μl; range 8–225.4 /μl). They were also markedly low in patients with hematological malignancies (median 168 /μl; range 48.4–330 /μl), showing no statistically significant difference between the two groups (*p* = 0.07; Fig. 3A). Among the patients with HIV infection, longitudinal CD4 + T cell counts had been assessed in five of nine patients. A gradual increase in CD4 + T cell counts was observed in these patients after the initiation of ART (Fig. 3B). IgG levels were assessed in all eight patients with hematological malignancies and eight of nine patients with HIV infection. The IgG levels in patients with hematological malignancies (median 643.5 mg/dl; range 205–2478 mg/dl) were significantly lower compared to those in patients with HIV infection (median 1691.5 mg/dl; range 1304–3014 mg/dl) (*p* < 0.01) (Fig. 3C).

### Treatments and outcomes

#### PML in patients with hematological malignancies

Six of eight patients could not receive any specific treatment for PML (Table 3). In Case M4, mirtazapine was continued until the development of dysphasia, which prevented her from taking it orally. In Case M8 (MM), mefloquine and mirtazapine were started. Seven of eight patients died with PML progression. The MM patient (Case M8) survived for 736 days after the onset of PML; the precise cause of death was unknown. The median times from onset and from diagnosis to death were 136 (range 25–736) days and 92 (range 22–718) days, respectively.

#### PML in patients with HIV infection

ART was started in all cases (Table 4). In Case H1, mefloquine was concomitantly administered. During treatment, three patients (Cases H4, H5, and H7) developed immune reconstitution inflammatory syndrome which resolved with corticosteroid treatment. Case H1 died of hemorrhagic shock caused by cytomegalovirus colitis and sepsis 13 days after the diagnosis of PML. Case H4 died of PML progression 72 days after the diagnosis. The remaining seven cases were alive at the last follow-up. Among them, four patients (including Case H2 and H9, who had negative CSF test results and were diagnosed with “possible PML”) showed improvement in their brain lesions and neurological symptoms ameliorated following the initiation of ART. Other three patients survived without obvious amelioration of neurological symptoms of PML. For the surviving patients, the median times from onset and from diagnosis to the end of follow-up were 564 (range 75–3043) days and 438 (range 56–3023) days, respectively.

## Discussion

The incidence of PML in patients with hematological malignancies is increasing and attracting attention; however, comprehensive reports are scarce [10] [11]. PML in patients with hematological malignancies consequently results from severe immune system impairment caused by treatment for these diseases, and the immune system cannot recover immediately. On the other hand, PML in patients with HIV infection is an opportunistic infection caused by the disease itself and the immune system can recover with ART. The differences in the clinical course between patients with hematological malignancies and those with HIV infection in this study demonstrated the importance of immune response in the pathogenesis and progression of PML. In this study, the median time from onset to the diagnosis of PML in patients with hematological malignancies was 33 days. In several cases, PML was not initially suspected, and some patients received additional chemotherapy for presumed CNS invasion of the malignancies, which may have accelerated the progression of PML. Therefore, we should be more cautious about the possibility of this rare neurological complication. Due to its rarity, it is often difficult to consider the possibility of PML for hematologists when patients exhibit neurological symptoms during or after treatment for hematological malignancies. When encountering a subacute progression pattern of neurological symptoms and typical MRI findings, an immediate CSF test, including JCV PCR, will contribute to a faster diagnosis. Earlier consultation with specialized radiologists and neurologists is also important because they are much more familiar with this rare neurological complication.

In the U.S. Food and Drug Administration “black box” warnings for PML, rituximab is categorized as Class 2, which indicates an association with a risk of developing PML [12]. Six patients with hematological malignancies in our study had received treatments containing anti-CD20 antibodies. Two received anti-CD20 antibodies combined with bendamustine. The risk of PML development has been reported to be potentially augmented by the combination of bendamustine and rituximab [13]. In the post-authorization report from the European Medicines Agency, 31 of 111 PML patients with CLL and 33 of 215 PML patients with non-Hodgkin B cell lymphoma had received combination therapy with bendamustine and rituximab [14]. Concomitant administration of bendamustine with rituximab has been reported to induce lower CD4 + T cell counts relative to R-CHOP therapy [15]. Therefore, it may be crucial to consider the risk of PML in patients treated with this combination therapy.

In this study, one patient developed PML after CAR-T cell therapy. Registry data from the Center for International Blood & Marrow Transplant Research revealed that the incidence of PML after CAR-T cell therapy was 0.9 per 1,000 patients [16]. Furthermore, in a phase I/II clinical study (GCT3013-01) of epcoritamab (a bispecific monoclonal antibody targeting CD3 and CD20), 2 of 157 patients (1.3%) with non-Hodgkin’s lymphoma developed PML during the observation period [17]. The present study also included a rare case of PML in a patient with MM. Although reports of PML in MM are limited, a report of a series of 6 cases indicated increased risk in patients treated long-term with immunomodulatory agents and highlighted the risk associated with bispecific antibodies [18]. The frequency of PML in patients treated with these newly introduced drugs and cellular therapies cannot be ignored. Because these agents are used for relapsed or refractory disease, we should consider the cumulative risk of PML in patients with a long treatment history.

The underlying mechanisms and risk factors for the development of PML remain incompletely elucidated. The cellular immune response plays a central role in preventing the development of PML, and CD4 + T cells are presumed to be particularly important for immune surveillance against JCV [19–22]. Low CD4 + T cell counts were generally observed in our patients with HIV infection, as is typical in this population. However, most of our patients with hematological malignancies also exhibited low CD4 + T cell counts at the diagnosis of PML, which were not significantly different from those in patients with HIV infection. Therefore, monitoring of CD4 + T cell counts may be useful for assessing the risk of PML in patients with hematological malignancies. Generally, hypergammaglobulinemia is observed in HIV-infected individuals; however, their antigen-specific immunity is reported to be impaired [23]. Furthermore, the humoral immune response is not considered sufficient to suppress the pathogenesis of PML [24]. Therefore, the significantly higher IgG levels in patients with HIV infection in our study do not seem to contribute to their better prognosis. Recently, the genetic susceptibility to PML was analyzed in a study of 110 drug-exposed PML cases compared with 718 drug-exposed matched controls [25]. Specific variants linked to the immune response were associated with the development of drug-induced PML. Genetic testing may become a useful tool for assessing the risk of PML in future.

In patients with HIV infection, CD4 + T cell counts gradually increased after the initiation of ART, indicating the importance of immune recovery. In drug-induced PML, discontinuation of the related drugs and recovery from the immunocompromised state are critical, as no specific antiviral drugs are effective against JCV. However, it is usually difficult to recover from a deep and prolonged immunocompromised state after the treatment of hematological malignancies, and the prognosis is dismal. Almost all patients with hematological malignancies in our study died of PML progression, with a median time of 92 days from the diagnosis to death, which is compatible with previous reports [10, 26]. Most of our patients with hematological malignancies could not receive any specific treatment for PML because the symptoms of PML had already progressed at the time of the diagnosis. Mirtazapine, a 5-HT_2A_ receptor antagonist, has been expected to be effective for PML because JCV enters oligodendrocytes through this receptor [27]. In our study, two patients with hematological malignancies received mirtazapine, but its efficacy was unclear. Previous studies reported its efficacy only in cases of multiple sclerosis (MS) treated with natalizumab [28]. Mefloquine is an antimalarial drug that has been tested for the treatment of PML. In our study, one patient with HIV infection was treated with mefloquine in combination with ART. However, no obvious suppressive effect against JCV was shown in an open-label, randomized control study using mefloquine for PML [29]. Immune checkpoint inhibitors are currently the most promising agents for the treatment of PML. In the Natural History Study of PML and the Inflammatory and Infectious Diseases of the Nervous System Study by the National Institutes of Health, eight patients with PML were treated with pembrolizumab; five of them showed clinical improvement or stabilization of PML accompanied by a reduction in the JCV load in the CSF and an increase of in vitro CD4 + and CD8 + anti-JCV activities [30]. A retrospective multicenter study from France reported that 79 patients with PML, including 38 with hematological malignancies, were treated with immune checkpoint inhibitors, and the one-year survival rate was 51.9% [31]. Importantly, the one-year survival rate remained at 50% even when analyzing only patients with hematological malignancies. This indicates that the immune response to JCV is crucial to suppress the progression of PML.

Considering the lack of an established treatment strategy for PML, it would be ideal to prevent its development. Natalizumab for relapsing–remitting MS is well known to be strongly associated with an increased risk of PML. Risk factors for PML in patients receiving natalizumab treatment have been clarified (32, 33) , and routine workups to check JCV antibody titers and periodic brain MRI are recommended. Although the incidence of PML is substantially lower in patients with hematological malignancies than in those with multiple sclerosis, identifying risk factors and implementing stringent surveillance may be required in future, particularly when treating hematological disorders with antibody drugs and cellular therapies. Furthermore, earlier suspicion of PML is important to prevent misdiagnoses or delayed diagnoses, which can exacerbate the progression of neurological symptoms.

This study had several limitations. Due to the small number of cases and the heterogeneity of patients with hematological malignancies, definitive risk factors or characteristics of PML could not be identified. Furthermore, because of the retrospective design of this study, some clinical data were missing in some cases. In addition, the diagnosis of PML was uncertain in some patients who were diagnosed with “possible PML” without positive CSF test results. Importantly, direct comparison of the prognosis of PML between patients with hematological malignancies and those with HIV infection is not reasonable, because immune reconstitution by ART significantly contributes to a better prognosis in patients with HIV infection. Therefore, we used HIV infection cohort as a reference group to elucidate the characteristics of PML in patients with hematological malignancies.

In conclusion, the immune response appears to be critical to overcoming the high mortality of PML. Greater awareness of PML may be warranted in patients with hematological malignancies to ensure an accurate diagnosis. The development of preventive and therapeutic strategies for PML would be essential in future.

## Data Availability

The data that support the findings of this study are available on request from the corresponding author, E.Y. The data are not publicly available due to their containing information that could compromise the privacy of research participants.
